# 1-Cyclo­pentyl­idene-2-(2,4-dinitro­phenyl)­hydrazine

**DOI:** 10.1107/S1600536808033345

**Published:** 2008-10-18

**Authors:** Ning-Ning Ji, Zhi-Qiang Shi

**Affiliations:** a Department of Chemistry, Taishan University, 271021 Taian, Shandong, People’s Republic of China; b Department of Materials Science and Chemical Engineering, Taishan University, 271021 Taian, Shandong, People’s Republic of China

## Abstract

The title compound, C_11_H_12_N_4_O_4_, was synthesized by the reaction of (2,4-dinitro­phen­yl)hydrazine with cyclo­penta­none. The cyclo­pentyl fragment is disordered over two sites with occupancies of 0.63 (1) and 0.37 (1). An intra­molecular N—H⋯O hydrogen bond helps to establish the conformation. Pairs of mol­ecules are held together by π–π inter­actions between adjacent benzene rings [centroid-to-centroid distance 3.589 (2) Å].

## Related literature

For background literature on Schiff bases, see: Liang (2007 ▶). For information on the properties of dinitro­phenyl­hydrazones, see: Baughman *et al.* (2004 ▶); Zare *et al.* (2005 ▶); El-Seify & El-Dossoki (2006 ▶); Kim & Yoon (1998 ▶). For bond-length data, see: Allen *et al.* (1987 ▶); Allen (2002 ▶).
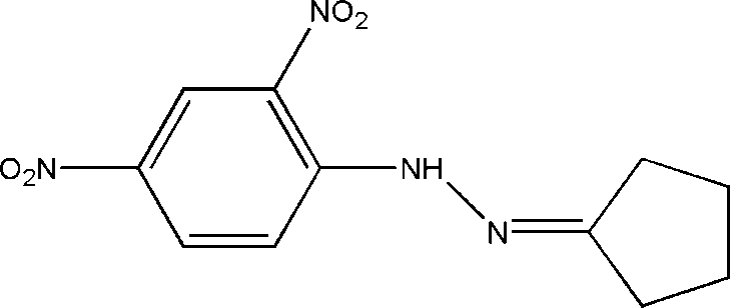


## Experimental

### 

#### Crystal data

C_11_H_12_N_4_O_4_
*M* *_r_* = 264.25
Monoclinic, 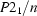

*a* = 6.962 (3) Å
*b* = 21.840 (10) Å
*c* = 8.162 (4) Å
β = 98.528 (9)°
*V* = 1227.3 (10) Å^3^
*Z* = 4
Mo *K*α radiation
μ = 0.11 mm^−1^
*T* = 295 (2) K
0.15 × 0.10 × 0.06 mm

#### Data collection

Bruker SMART CCD diffractometer
Absorption correction: multi-scan (*SADABS*; Sheldrick, 1996 ▶) *T*_min_ = 0.984, *T*_max_ = 0.993
6423 measured reflections
2168 independent reflections
1353 reflections with *I* > 2σ(*I*)
*R*_int_ = 0.029

#### Refinement

*R*[*F* ^2^ > 2σ(*F* ^2^)] = 0.042
*wR*(*F* ^2^) = 0.129
*S* = 1.02
2168 reflections
192 parameters
H-atom parameters constrained
Δρ_max_ = 0.13 e Å^−3^
Δρ_min_ = −0.14 e Å^−3^

### 

Data collection: *SMART* (Bruker, 2003 ▶); cell refinement: *SAINT* (Bruker, 2003 ▶); data reduction: *SAINT*; program(s) used to solve structure: *SHELXS97* (Sheldrick, 2008 ▶); program(s) used to refine structure: *SHELXL97* (Sheldrick, 2008 ▶); molecular graphics: *SHELXTL* (Sheldrick, 2008 ▶); software used to prepare material for publication: *SHELXTL*.

## Supplementary Material

Crystal structure: contains datablocks global, I. DOI: 10.1107/S1600536808033345/fb2114sup1.cif

Structure factors: contains datablocks I. DOI: 10.1107/S1600536808033345/fb2114Isup2.hkl

Additional supplementary materials:  crystallographic information; 3D view; checkCIF report

## Figures and Tables

**Table 1:** Hydrogen-bond geometry (Å, °)

*D*—H⋯*A*	*D*—H	H⋯*A*	*D*⋯*A*	*D*—H⋯*A*
N3—H3⋯O2	0.86	1.99	2.605 (2)	128

**Fig. 1.**
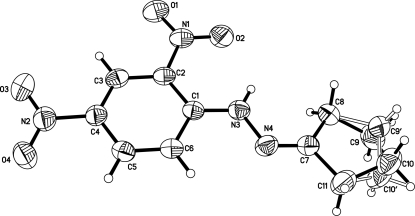
The title molecular with displacement ellipsoids drawn at the 50% probability level.

**Fig. 2.**
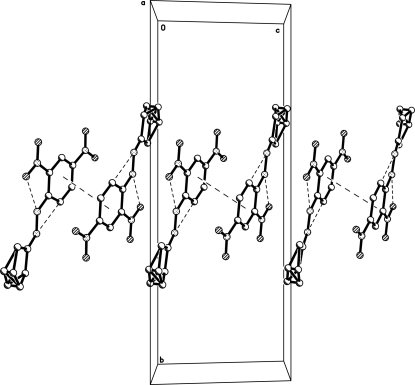
The view of the structure. The dashed lines indicate the N—H···O hydrogen bonds and C—H···N interactions as well as π–π ring electron interactions.

## supplementary crystallographic information

### Comment

Schiff bases and their complexes are widely used in the fields of biology, catalysis *etc.* (Liang, 2007). Especially, the dinitrophenylhydrazones exhibit good nonlinear optical (NLO) and crystalline properties (Baughman *et al.*, 2004). The benzophenone-2,4-dinitrophenylhydrazone derivatives are important because of their significant molecular nonlinearities and remarkable ability to crystallize in non-centrosymmetric crystal systems (Zare *et al.*, 2005; El-Seify & El-Dossoki, 2006; Kim & Yoon, 1998). In order to search for new dinitrophenylhydrazones, the title compound was synthesized and its crystal structure is reported here (Fig. 1). The obtained unrestrained bond lengths and angles are in good agreement with the expected values (Allen *et al.*, 1987) in the non-disordered region. In the crystal structure (Fig. 2), the molecules are stabilized by N—H···O hydrogen bonds (Table 1), C—H···N interactions (C6—H6···N4: 0.93, 2.39, 2.722 (3) Å and 101.1°) and by π–π electron interactions between the benzene rings. The distances between the centroids of the stacked benzene rings are 3.589 (2) Å though the molecules are situated in rather equidistant planes.

### Experimental

The title compound was synthesized by the reaction of (2,4-dinitro-phenyl)-hydrazine (1 mmol, 198.1 mg) with cyclopentanone (1 mmol, 84.1 mg) in ethanol (30 ml) under reflux conditions (348 K) for 3 h. The solvent was removed and the solid product was recrystallized from tetrahydrofuran. Brown crystals that were suitable for X-ray diffraction study were grown in the course of three days. Yield, 227.2 mg, 86%; m. p. 318–320 K.

Analysis calculated for C_11_H_12_N_4_O_4_: C 50.00, H 4.58, N 21.20%; found: C 49.97, H 4.52, N 21.15%.

### Refinement

All the H atoms except those attached to the disordered atoms C9, C9', C10 and C10' could have been distinguished in the difference electron density maps. During the refinement the H atoms were situated into idealized positions, constrained and refined as riding atoms. The constraints: C_aryl_—H = 0.93; C_methylene_—H 0.97 Å, N—H = 0.86 Å; *U*_iso_(H) = 1.2*U*_eq_(carrier atom). The disorder was treated with the following restraints: The distances C9—C10 and C9'—C10' were restrained to 1.485 (10) Å, the distances C8—C9, C8—C9', C10—C11 and C10'—C11 to 1.520 (10) Å and the distances C7—C8, C7—C11 to 1.503 (10) Å. The values of these distances were retrieved from the Cambridge Crystal Structure Database (version 5.29 plus updates to January 2008; Allen, 2002) for the structures that contained the fragment —NH—N ═cyclopentyl that is present in the title structure. The retrieved structures HULJON, KERWUA, NAQSAZ and RAKHUH are without disorder, errors and with the *R*-factor < 0.05. The displacement parameters of the atoms C9', C10', C9' and C10' were restrained by the command SIMU with the default parameters (0.04, 0.08, 1.7) of the refinement program SHELXL97 (Sheldrick, 2008).

### Crystal data

**Table:**

C_11_H_12_N_4_O_4_	*F*(000) = 552
*M**_r_* = 264.25	*D*_x_ = 1.430 Mg m^−^^3^
Monoclinic, *P*2_1_/*n*	Melting point = 318–320 K
Hall symbol: -P 2yn	Mo *K*α radiation, λ = 0.71073 Å
*a* = 6.962 (3) Å	Cell parameters from 1213 reflections
*b* = 21.84 (1) Å	θ = 3.1–21.3°
*c* = 8.162 (4) Å	µ = 0.11 mm^−^^1^
β = 98.528 (9)°	*T* = 295 K
*V* = 1227.3 (10) Å^3^	Block, brown
*Z* = 4	0.15 × 0.10 × 0.06 mm

### Data collection

**Table:**

Bruker SMART CCD diffractometer	2168 independent reflections
Radiation source: fine-focus sealed tube	1353 reflections with *I* > 2σ(*I*)
graphite	*R*_int_ = 0.029
φ and ω scans	θ_max_ = 25.0°, θ_min_ = 1.9°
Absorption correction: multi-scan (SADABS; Sheldrick, 1996)	*h* = −7→8
*T*_min_ = 0.984, *T*_max_ = 0.993	*k* = −20→26
6423 measured reflections	*l* = −9→9

### Refinement

**Table:**

Refinement on *F*^2^	Secondary atom site location: difference Fourier map
Least-squares matrix: full	Hydrogen site location: difference Fourier map
*R*[*F*^2^ > 2σ(*F*^2^)] = 0.042	H-atom parameters constrained
*wR*(*F*^2^) = 0.129	*w* = 1/[σ^2^(*F*_o_^2^) + (0.0582*P*)^2^ + 0.1369*P*] where *P* = (*F*_o_^2^ + 2*F*_c_^2^)/3
*S* = 1.02	(Δ/σ)_max_ < 0.001
2168 reflections	Δρ_max_ = 0.13 e Å^−^^3^
192 parameters	Δρ_min_ = −0.14 e Å^−^^3^
0 restraints	Extinction correction: SHELXL97 (Sheldrick, 2008), Fc^*^=kFc[1+0.001xFc^2^λ^3^/sin(2θ)]^-1/4^
64 constraints	Extinction coefficient: 0.008 (2)
Primary atom site location: structure-invariant direct methods	

### Special details

**Table:**

Geometry. All e.s.d.'s (except the e.s.d. in the dihedral angle between two l.s. planes) are estimated using the full covariance matrix. The cell e.s.d.'s are taken into account individually in the estimation of e.s.d.'s in distances, angles and torsion angles; correlations between e.s.d.'s in cell parameters are only used when they are defined by crystal symmetry. An approximate (isotropic) treatment of cell e.s.d.'s is used for estimating e.s.d.'s involving l.s. planes.
Refinement. Refinement of *F*^2^ against ALL reflections. The weighted *R*-factor *wR* and goodness of fit *S* are based on *F*^2^, conventional *R*-factors *R* are based on *F*, with *F* set to zero for negative *F*^2^. The threshold expression of *F*^2^ > σ(*F*^2^) is used only for calculating *R*-factors(gt) *etc*. and is not relevant to the choice of reflections for refinement. *R*-factors based on *F*^2^ are statistically about twice as large as those based on *F*, and *R*- factors based on ALL data will be even larger.

### Fractional atomic coordinates and isotropic or equivalent isotropic displacement parameters (Å^2^)

**Table:**

	*x*	*y*	*z*	*U*_iso_*/*U*_eq_	Occ. (<1)
O1	0.7332 (3)	−0.13258 (8)	0.3023 (2)	0.0970 (6)	
O2	0.6598 (2)	−0.04170 (8)	0.3749 (2)	0.0883 (6)	
O3	1.2739 (3)	−0.17276 (10)	0.0323 (3)	0.1106 (7)	
O4	1.4641 (3)	−0.09968 (8)	−0.0180 (2)	0.0979 (6)	
N1	0.7661 (3)	−0.07782 (10)	0.3142 (2)	0.0715 (5)	
N2	1.3231 (3)	−0.11920 (11)	0.0400 (2)	0.0802 (6)	
N3	0.8813 (2)	0.05130 (8)	0.3322 (2)	0.0643 (5)	
H3	0.7731	0.0416	0.3643	0.077*	
N4	0.9487 (3)	0.11105 (8)	0.3468 (2)	0.0716 (5)	
C1	0.9877 (3)	0.00874 (9)	0.2673 (2)	0.0563 (5)	
C2	0.9367 (3)	−0.05376 (9)	0.2535 (2)	0.0583 (5)	
C3	1.0473 (3)	−0.09546 (10)	0.1801 (2)	0.0641 (6)	
H3A	1.0114	−0.1365	0.1717	0.077*	
C4	1.2096 (3)	−0.07577 (10)	0.1204 (2)	0.0634 (6)	
C5	1.2658 (3)	−0.01508 (10)	0.1343 (3)	0.0653 (6)	
H5	1.3777	−0.0024	0.0944	0.078*	
C6	1.1586 (3)	0.02600 (10)	0.2058 (3)	0.0631 (6)	
H6	1.1986	0.0666	0.2145	0.076*	
C7	0.8305 (3)	0.15004 (10)	0.3923 (3)	0.0690 (6)	
C8	0.6290 (3)	0.14145 (10)	0.4287 (3)	0.0785 (7)	
H8A	0.6292	0.1186	0.5306	0.094*	
H8B	0.5503	0.1197	0.3391	0.094*	
C9	0.5515 (8)	0.2067 (3)	0.4458 (10)	0.0954 (16)	0.631 (10)
H9A	0.4572	0.2078	0.5221	0.114*	0.631 (10)
H9B	0.4921	0.2227	0.3393	0.114*	0.631 (10)
C10	0.7337 (8)	0.2425 (3)	0.5134 (10)	0.0972 (15)	0.631 (10)
H10A	0.7157	0.2860	0.4924	0.117*	0.631 (10)
H10B	0.7690	0.2360	0.6316	0.117*	0.631 (10)
C10'	0.7036 (14)	0.2469 (4)	0.4218 (16)	0.090 (2)	0.369 (10)
H10C	0.6345	0.2536	0.3111	0.108*	0.369 (10)
H10D	0.7224	0.2859	0.4788	0.108*	0.369 (10)
C9'	0.5961 (19)	0.2020 (4)	0.5176 (14)	0.087 (2)	0.369 (10)
H9'1	0.4591	0.2119	0.5080	0.105*	0.369 (10)
H9'2	0.6518	0.2006	0.6337	0.105*	0.369 (10)
C11	0.8888 (4)	0.21600 (11)	0.4170 (4)	0.0979 (8)	
H11A	0.8868	0.2367	0.3116	0.117*	
H11B	1.0176	0.2196	0.4805	0.117*	

### Atomic displacement parameters (Å^2^)

**Table:**

	*U*^11^	*U*^22^	*U*^33^	*U*^12^	*U*^13^	*U*^23^
O1	0.0998 (14)	0.0748 (12)	0.1191 (15)	−0.0291 (10)	0.0247 (11)	0.0028 (10)
O2	0.0678 (11)	0.0882 (12)	0.1131 (14)	−0.0079 (9)	0.0276 (10)	0.0071 (10)
O3	0.1313 (17)	0.0738 (12)	0.1310 (17)	0.0164 (11)	0.0340 (13)	−0.0039 (11)
O4	0.0988 (14)	0.1102 (14)	0.0911 (13)	0.0162 (11)	0.0353 (11)	0.0015 (11)
N1	0.0660 (13)	0.0744 (14)	0.0725 (13)	−0.0132 (11)	0.0047 (10)	0.0104 (10)
N2	0.0869 (15)	0.0837 (16)	0.0694 (13)	0.0160 (13)	0.0095 (11)	0.0062 (11)
N3	0.0584 (11)	0.0658 (11)	0.0695 (12)	−0.0059 (9)	0.0124 (9)	0.0045 (9)
N4	0.0675 (12)	0.0604 (11)	0.0879 (14)	−0.0060 (10)	0.0151 (10)	0.0050 (10)
C1	0.0544 (12)	0.0634 (12)	0.0494 (11)	−0.0002 (10)	0.0019 (9)	0.0087 (9)
C2	0.0562 (12)	0.0630 (12)	0.0535 (11)	−0.0059 (10)	0.0011 (9)	0.0108 (10)
C3	0.0697 (14)	0.0613 (13)	0.0575 (12)	−0.0040 (11)	−0.0025 (11)	0.0099 (10)
C4	0.0685 (14)	0.0646 (13)	0.0555 (12)	0.0088 (11)	0.0032 (10)	0.0091 (10)
C5	0.0593 (12)	0.0761 (14)	0.0610 (13)	−0.0003 (11)	0.0104 (10)	0.0106 (11)
C6	0.0625 (13)	0.0638 (13)	0.0626 (13)	−0.0067 (10)	0.0080 (11)	0.0077 (10)
C7	0.0733 (14)	0.0660 (13)	0.0688 (14)	−0.0015 (12)	0.0145 (11)	0.0070 (11)
C8	0.0805 (16)	0.0807 (15)	0.0784 (15)	−0.0003 (12)	0.0254 (12)	0.0018 (12)
C9	0.104 (3)	0.095 (3)	0.093 (3)	0.023 (2)	0.030 (3)	0.011 (3)
C10	0.118 (3)	0.076 (3)	0.098 (3)	0.011 (2)	0.016 (3)	0.002 (3)
C10'	0.110 (4)	0.066 (3)	0.094 (4)	0.012 (3)	0.016 (4)	0.007 (4)
C9'	0.095 (4)	0.080 (3)	0.090 (4)	0.010 (3)	0.026 (4)	0.007 (4)
C11	0.1034 (19)	0.0699 (15)	0.123 (2)	−0.0041 (14)	0.0257 (16)	0.0030 (14)

### Geometric parameters (Å, °)

**Table:**

O1—N1	1.219 (2)	C7—C11	1.502 (3)
O2—N1	1.234 (2)	C8—C9	1.538 (6)
O3—N2	1.218 (2)	C8—C9'	1.542 (10)
O4—N2	1.227 (3)	C8—H8A	0.9700
N1—C2	1.452 (3)	C8—H8B	0.9700
N2—C4	1.453 (3)	C9—C10	1.522 (7)
N3—C1	1.345 (3)	C9—H9A	0.9700
N3—N4	1.386 (2)	C9—H9B	0.9700
N3—H3	0.8600	C10—C11	1.540 (6)
N4—C7	1.277 (3)	C10—H10A	0.9700
C1—C6	1.409 (3)	C10—H10B	0.9700
C1—C2	1.411 (3)	C10'—C11	1.461 (9)
C2—C3	1.385 (3)	C10'—C9'	1.519 (2)
C3—C4	1.364 (3)	C10'—H10C	0.9700
C3—H3A	0.9300	C10'—H10D	0.9700
C4—C5	1.382 (3)	C9'—H9'1	0.9700
C5—C6	1.354 (3)	C9'—H9'2	0.9700
C5—H5	0.9300	C11—H11A	0.9700
C6—H6	0.9300	C11—H11B	0.9700
C7—C8	1.488 (3)		
			
O1—N1—O2	122.8 (2)	C9—C8—H8B	110.8
O1—N1—C2	118.8 (2)	C9'—C8—H8B	131.9
O2—N1—C2	118.38 (19)	H8A—C8—H8B	108.9
O3—N2—O4	123.3 (2)	C8—C9—C10	102.9 (5)
O3—N2—C4	118.9 (2)	C8—C9—H9A	111.2
O4—N2—C4	117.9 (2)	C10—C9—H9A	111.2
C1—N3—N4	119.05 (18)	C8—C9—H9B	111.2
C1—N3—H3	120.5	C10—C9—H9B	111.2
N4—N3—H3	120.5	H9A—C9—H9B	109.1
C7—N4—N3	115.38 (19)	C11—C10—C9	103.4 (5)
N3—C1—C6	119.90 (19)	C11—C10—H10A	111.1
N3—C1—C2	123.60 (19)	C9—C10—H10A	111.1
C6—C1—C2	116.5 (2)	C11—C10—H10B	111.1
C3—C2—C1	121.43 (19)	C9—C10—H10B	111.1
C3—C2—N1	116.4 (2)	H10A—C10—H10B	109.0
C1—C2—N1	122.1 (2)	C11—C10'—C9'	102.7 (7)
C4—C3—C2	119.3 (2)	C11—C10'—H10C	111.2
C4—C3—H3A	120.3	C9'—C10'—H10C	111.2
C2—C3—H3A	120.3	C11—C10'—H10D	111.2
C3—C4—C5	120.8 (2)	C9'—C10'—H10D	111.2
C3—C4—N2	119.4 (2)	H10C—C10'—H10D	109.1
C5—C4—N2	119.8 (2)	C10'—C9'—C8	101.1 (7)
C6—C5—C4	120.2 (2)	C10'—C9'—H9'1	111.5
C6—C5—H5	119.9	C8—C9'—H9'1	111.5
C4—C5—H5	119.9	C10'—C9'—H9'2	111.5
C5—C6—C1	121.7 (2)	C8—C9'—H9'2	111.5
C5—C6—H6	119.2	H9'1—C9'—H9'2	109.4
C1—C6—H6	119.2	C10'—C11—C7	103.0 (4)
N4—C7—C8	129.9 (2)	C7—C11—C10	103.5 (3)
N4—C7—C11	120.4 (2)	C10'—C11—H11A	85.0
C8—C7—C11	109.8 (2)	C7—C11—H11A	111.1
C7—C8—C9	104.8 (3)	C10—C11—H11A	111.1
C7—C8—C9'	101.3 (4)	C10'—C11—H11B	134.2
C7—C8—H8A	110.8	C7—C11—H11B	111.1
C9—C8—H8A	110.8	C10—C11—H11B	111.1
C9'—C8—H8A	91.1	H11A—C11—H11B	109.0
C7—C8—H8B	110.8		

### Hydrogen-bond geometry (Å, °)

**Table:**

*D*—H···*A*	*D*—H	H···*A*	*D*···*A*	*D*—H···*A*
N3—H3···O2	0.86	1.99	2.605 (2)	128
